# *Nannizziopsis arthrosporioides* infection mimicking ophidiomycosis in ball pythons (*Python regius*)

**DOI:** 10.1016/j.mmcr.2025.100733

**Published:** 2025-09-13

**Authors:** Krista A. Keller, Laura Adamovicz, Cathy Johnson-Delaney, Karen A. Terio

**Affiliations:** a Department of Medicine and Epidemiology, School of Veterinary Medicine, University of California, Davis, CA, 95616, USA; b Wildlife Epidemiology Laboratory, College of Veterinary Medicine, University of Illinois, Urbana, IL, 61802, USA; c Northwest Zoological Supply, LLC, WA, Monroe, 98201, USA; d Zoological Pathology Program, College of Veterinary Medicine, University of Illinois, Brookfield, IL, 60513, USA

**Keywords:** Nannizziopsis, Onygenales, Dermatomycosis, Squamate, Fungus

## Abstract

A wild caught snake presented with progressive dermatopathy and was euthanized due to a clinical suspicion for ophiomycosis. Over the next 7 days, six additional ball pythons, maintained in the same room but in separate cages from the index case developed similar progressive dermatomycoses and were euthanized. Histopathologic evaluation showed dermal granulomas with intralesional fungi and DNA from the lesions clustered in a monophyletic group with other isolates of *N. athrosporioides*. Colony and microscopic morphology of fungus from these cases matched published descriptions of *N. arthrosporioides*. While ophidiomycosis may be a major differential for dermatomycosis in snakes, molecular confirmation should be pursued, as additional fungi may cause similar lesions in this taxon.

## 1 Introduction

The fungal genus *Nannizziopsis* includes several keratinophillic fungi that infect both reptiles and humans. Previously grouped and published under the nomenclature *Chrysosporium* anamorph of *Nannizziopsis vriesii* (CANV), molecular sequencing has separated morphologically distinct species into separate genera within Order Onygenales including *Nannizziopsis*, *Paranannizziopsis*, and *Ophidiomyces* [1,2]. *Nannizziopsis* infected reptiles present with clinical signs associated with dermatomycosis (dermal ulceration and crusting) and systemic infection has been reported in the presence and absence of cutaneous infection [[3], [4], [5], [6]]. Early literature noted a strong tendency towards host pathogen relationships with *Nannizziopsis* spp. typically infecting lizards and *Ophidiomyces ophidiicola* infecting snakes. However, recent experimental work has shown that *N. guarroi*, the causative agent of “yellow fungus disease” of bearded dragons, can infect snakes and *O. ophidiicola*, the causative agent of “snake fungal disease” can cause infection in lizards [6]. Further, recent reports have identified *N. arthrosporioides* infection in chelonians [7,8], indicating that wider host pathogen relationships should be considered.

This case series reports on the postmortem histologic, fungal morphologic, and molecular confirmation of *N. arthrosporioides* infection in a group of ball pythons (*Python regius*) that were housed in the same room as another infected snake. While natural *Nannizziopsis* infection has been reported in snakes [9], based upon a Google Scholar and Pubmed search on September 10th, 2025, there are no reports of *N. arthrosporioides* infection in snakes in the literature. Due to the similar clinical manifestations of infection between the genera previously grouped under CANV, molecular diagnostics should be pursued to determine etiologic agents responsible for dermatomycoses of reptiles. Further, although not previously described, *N. arthrosporiodes* should be considered as a differential for snakes with dermatomycosis.

## 2 Case presentation

### 2.1 Index case: central American boa

A wild caught juvenile Central American boa (*Boa imperator*) was noted upon examination (Day 0) to have multifocal ulcerative skin lesions along the entire length of its body; lesions were variable in shape and size, thickened, and associated with dermal crusts and ulceration. The snake had recently been imported to a breeding facility that housed multiple species of reptiles and was being maintained in a quarantine room with other recently imported individuals. The snake exhibited a low body condition, was infested with mites, and suffered from a right sided sub spectacular abscess. Based upon these findings, a decision for humane euthanasia was made. Given that this snake was a part of a larger collection of reptiles destined for the pet trade and an infectious cause, primarily *O. ophidiicola*, was suspected, a perimortem cutaneous swab was collected for submission for commercially available next generation sequencing that amplifies fungal DNA through ITS-2 targets and bacterial DNA through 16S rRNA V1–V3(NGS, MiDog LLC, 14762 Bentley Circle, Tustin, CA 92780).

After euthanasia, a gross necropsy was performed on site; internal viscera examined were grossly normal, and postmortem lesions were limited to the clinically described dermatomycosis. Postmortem samples of skin, heart and great vessels, lungs, liver, kidney, and intestine were collected into formalin for histopathologic analysis (Northwest ZooPath, Wonroe, WA, USA).

Evaluation of hematoxylin and eosin (H&E) stained skin samples reported epidermal hyperplasia, hyperkeratosis, crust formation, and ulceration associated with invasive hyphae within the examined skin samples. Granuloma formation was oriented around fungal elements and associated inflammation extended into the dermis. Based upon the fungal morphology in tissue and the host species, the primary clinical differential for the dermatomycosis was infection with *O ophidiicola*.

The commercial NGS analysis detected 2 fungal agents: *Nannizziopsis arthrosporioides* and *Ascomycota* sp. The former was noted in a relative fungal abundance of 99.85 % with an absolute abundance of 7400 cells/sample. Although dermatomycosis from *O. ophidiicola* was initially suspected based on signalment, clinical signs, and histopathologic evaluation, the lack of detection of DNA from this microbe and detection of *N. arthrosporioides* DNA supported a diagnosis of dermatomycosis attributable to *N. arthrospordioides*.

### 2.2 Subsequent cases: ball pythons

**Fig. 1:**
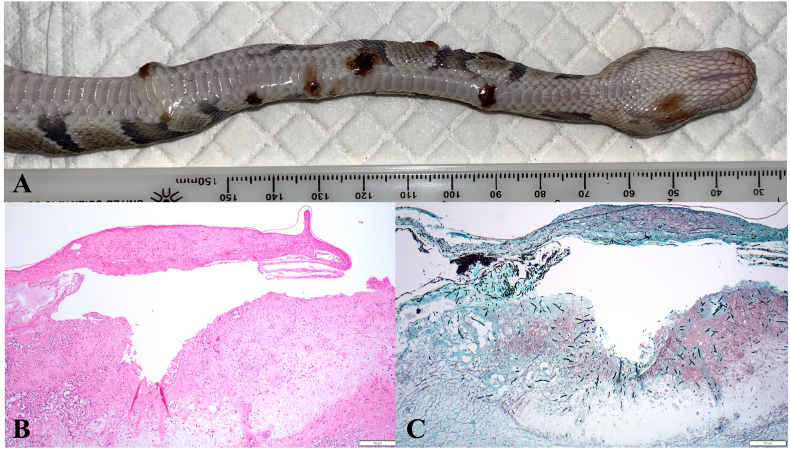
*Nannizziopsis arthrosporioides* infection in a ball python (*Python regius*). A – Multifocal, raised, red and crusty lesions are present in this young ball python, with similar lesions appearing in 5 other in contact ball pythons. Replicate sections of scaled skin stained with Hematoxylin-eosin (A) and Grocott's methenamine silver (GMS) (B). There is ulceration of the epithelium with serocellular crust overlying the exposed dermis. The dermis contains heterophils, cellular debris, and numerous argyrophilic 3–5 μm branching fungal hyphae.

Six juvenile (weight range 71–127 g) captive bred ball pythons kept in the same room but in separate cages from the index cage were noted to have similar skin lesions (multifocal thickened crusts, some with ulceration present, Fig. 1) exhibiting clinical progression within 7 days of the index case. On day 7, a pooled cutaneous swab was taken from the six snakes and submitted for the previously used commercial NGS service that confirmed detection of *N. arthrosporioides* (relative fungal abundance 99.5 %) without the detection of other fungal DNA. After receiving commercial NGS results and noting progression of the skin lesions, the six ball pythons were humanely euthanized to limit further infection spread to additional reptiles on the grounds. Swabs from the cutaneous lesions of 2 snakes were collected at the time of euthanasia and submitted for fungal culture. Carcasses were shipped frozen for necropsy and further evaluation.

**Fig. 2:**
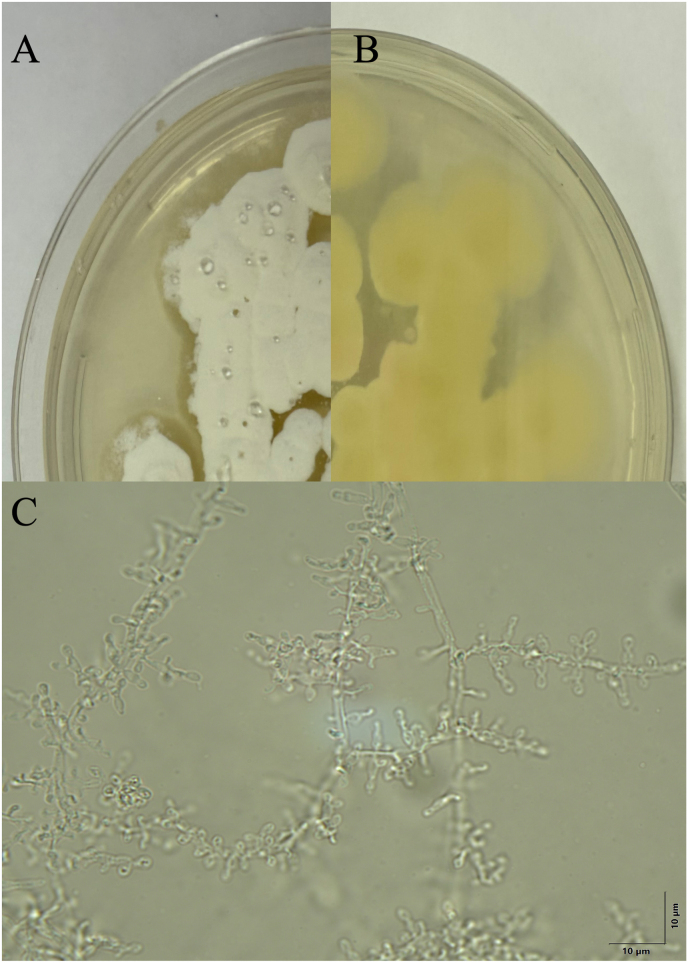
Colony morphology and direct microscopy of *Nannizziopsis arthrosporioides* pure growth on potato dextrose agar after 14 days of incubation. The circular to coalescing colonies are white and cottony on agar (A) and yellow colored on reverse (B). Direct microscopy of hyphae, 100X, unstained (C). Hyphae (1–3 μm in width) are septate, smooth walled, and branching with frequent fertile hyphae bearing sessile conidia.

To reduce cutaneous bacterial and saprophytic microbial growth, cutaneous swabs were streaked onto modified agar composed of potato dextrose agar (Remel, Thermo Scientific, Lenexa, KS, USA), penicillin-streptomycin (60,000/60 U/liter; Fisher Scientific), gentamicin sulfate (50 mg/L; Sigma-Aldrich), and cycloheximide (400 mg/L; Acros Organics) then incubated under ambient lighting at 23 °C (73.4 °F). After 10–14 days of incubation round to coalescing, white cottony colony growth morphologically consistent with *Nannizziopsis* spp. was present (Fig. 2A and B). Fungal elements were microscopically examined from colony tape preps. Arthroconidia, sessile conidia, and free conidia were noted and ranged in shape from pyriform, cylindrical and subglobose measuring 2.5–5 by 1.4–3 μm. Septated, smooth walled branching and sometimes undulating hyphae were present, measuring 1–3 μm in width (Fig. 2C).

Extraction of DNA from samples of pure culture material was performed using a commercially available kit (QIAamp DNA mini-Kit, Qiagen Inc., Valencia, CA, USA). Manufacturer's recommendations were followed with the addition of a 1-h incubation with 300U of lyticase (Sigma-Aldrich, St. Louis, MO, USA) at 37 °C (98.6°F) prior to the lysis step. PCR targets for fungal identification were selected to enable direct comparison to other Onygenalean fungi (Schtigel et al., 2013) and included the 18S-28S ribosomal internal transcribed spacer region (ITS) [10], D1/D2 domains of the 28S ribosomal DNA (D1/D2 rDNA) [11], the actin gene [12], and the β-tubulin gene [13]. Gel electrophoresis on 1 % agarose gel of PCR products was performed to confirm that successful DNA amplification occurred and to verify the expected size of the PCR product by comparison to a commercial DNA ladder included in a separate well. A portion of the PCR product (15uL) was used for gel electrophoresis. Following that, 5uL of the remaining PCR product (roughly 35uL of the original 50uL reaction remained following gel electrophoresis) was treated with ExoSAP-IT (ExoSAP-IT, USB Corporation, Cleveland, OH, USA), and submitted for commercial DNA sequencing (ACGT, Wheeling, IL, USA). Chromatograms were visually inspected and trimmed of primer sequences and low-quality base pair calls. Sequences from the fungal isolate have been deposited in GenBank (accession numbers: PV551206, PV551207, PV435306, PV420619).

**Fig. 3:**
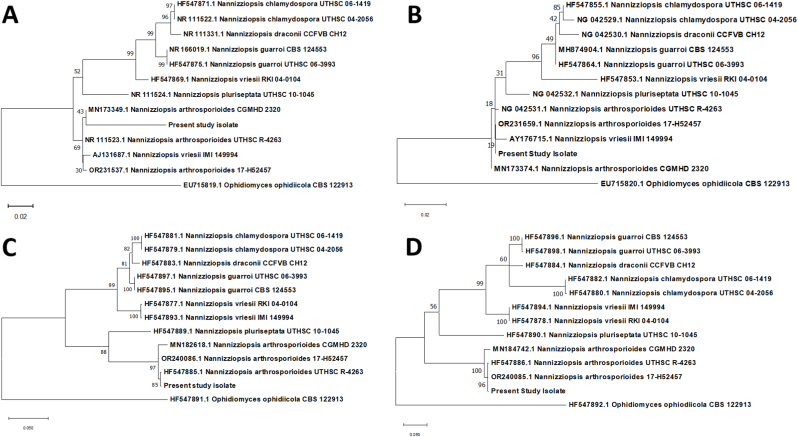
Phylogenetic trees showing genetic relationships between *Nannizziopsis arthrosporioides* causing dermatomycosis in ball pythons (*Python regius*) and GenBank referenced sequences that cause similar clinical lesions in reptiles. A – Internal transcription spacer, B – D1/D2 hypervariable region, C = actin, D = beta tubulin.

**Fig. 4:**
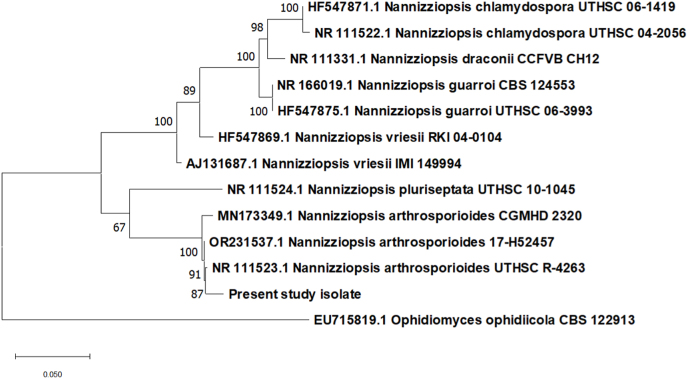
Concatenated phylogenetic tree showing that *Nannizziopsis arthrosporioides* isolated from dermatomycotic lesions in ball pythons (*Python regius*) show distinct monophyletic clustering with other isolates of *N. arthrosporioides* accessed from Genbank.

Phylogenetic analysis, concatenating all four gene targets, was performed using MEGA X [14] and homologous onygenalean DNA sequences obtained from GenBank, accessed on 9/5/2023. Sequence alignment was performed using MUSCLE with default parameters. The most parsimonious nucleotide substitution model was identified using the software's model selection algorithm. Evolutionary rate differences were modeled with a discrete gamma distribution, and all models were bootstrapped with 1000 random re-samplings of the data. Separate maximum likelihood phylogenetic trees were constructed for each gene target individually, and then for all targets concatenated. The fungal sequence clustered in well-supported monophyletic groups with *N. arthrosporioides* for the actin (97 % bootstrap support) and beta-tubulin (100 % bootstrap support) genes (Fig. 3). In contrast, for the D1/D2 rDNA and ITS targets, the fungal sequence clustered in poorly to moderately supported monophyletic groups including *N. arthrosporioides* and *N. vriesii* (18 % bootstrap support for D1/D2 rDNA, 69 % for ITS) (Fig. 3). In the concatenated analysis, the fungal sequence was clustered in a monophyletic group with *N. arthrosporioides* with high bootstrap support (100 %) (Fig. 4).

Postmortem examination was performed after carcasses were defrosted over 24 hours in refrigeration; gross lesions were limited to the clinically detected cutaneous lesions. Given the small size of each of the snakes, the entire carcass was collected in formalin after ventral midline incision. Tissues examined histologically included the trachea, esophagus, thyroid, heart, lung, great vessels, liver, kidney, spleen, gonads, stomach, intestine, pancreas, skeletal muscle, bone, brain and skin. Visceral lesions were limited to atrophy of fat and mild hepatocellular vacuolation in some snakes. Evidence of systemic inflammation was not present. Histopathologic appearance of H&E-stained skin samples from all snakes was similar and consisted of ulceration with superficial serocellular crusts and inflammation that extended into the underlying dermis and, in some cases, the underlying skeletal muscle (Fig. 1). On H&E-stained sections, non-staining 3–5-μm parallel walled hyphae were noted deep within cutaneous crusts. Skin sections stained with Grocott's methenamine silver stain (GMS) had argyrophilic 3-4microns wide, septate hyphae with rare branching. Additionally, 3 x 6-μm ovoid conidia were present in moderate numbers.

## 3 Discussion

This case series outlines the clinical findings of two species of snakes infected with *N. arthrosporioides,* exemplifying the contagious nature of this infection*.* Each snake presented with signs consistent with dermatomycosis including cutaneous crusting, discoloration, and ulcerations. After the initial detection of fungal organisms on histopathology from the index case, *O. ophidiicola*, the most common cause of dermatomycosis in snakes and the cause of “snake fungal disease” was clinically suspected [4,5]. Infection with *O. ophidiicola* has been documented in a wide variety of snake species in free-ranging and managed-care settings [4,5]. However, this case highlights the importance of molecular detection techniques to elucidate the identity of fungal organisms, as clinical signs and colony morphology alone is not enough to assign fungal genus or species of *Nannizziopsis, Paranannizziopsis* and *Ophidiomyces*.

*Nannizziopsis* spp. have been classically reported to infect saurians [4,5]. However, recent experimental trials have proven that *N. guarroi,* the cause of “yellow fungus disease” in bearded dragons, can infect corn snakes (*Pantherophis guttatus*) [6]. Further, a recent report potentially identified a novel *Nannizziopsis* species in free ranging snakes with dermal lesions in Taiwan [9]. Other investigators have detected *N. arthrosporioides* infection in an African side necked turtle (*Pelomedusa subrufa*), a 10.13039/100019832Galapagos tortoise (*Chelonoidis nigra*), and a human suffering from tongue infection [7,8,15]. The human infection was also initially misidentified as another fungus prior to molecular characterization [15]. These findings support that nannizziomycosis should be suspected in a wider host range than lizards and potentially include all reptiles, including snakes. Although the human case did not report contact with reptiles, this fungi should be considered zoonotic [15].

The snakes presented here represent natural *N. arthrosporioides* infection of two non-lacertian species. It is suspected that the index case brought the fungus into the facility and the ball pythons, housed in the same quarantine room, were exposed, as they had previously been noted to lack skin lesions. The initial point of infection for the index case was unable to be determined. Based upon the poor clinical state of the boa at the time of diagnosis, the host immune system was likely compromised either secondary to the mycosis or as a predisposing factor to the development of the mycosis. Given the recent import and travel of this individual, environmental stressors that may precipitate immunosuppression must also be considered. Further work is required to better understand the host:microbe:environment triad of infection as it relates to *Nannizziopsis* spp. infections in reptiles.

## CRediT authorship contribution statement

**Krista A. Keller:** Writing – review & editing, Writing – original draft, Supervision, Resources, Formal analysis. **Laura Adamovicz:** Writing – review & editing, Writing – original draft, Visualization, Validation, Methodology, Investigation, Formal analysis. **Cathy Johnson-Delaney:** Writing – review & editing, Investigation. **Karen A. Terio:** Writing – review & editing, Visualization, Methodology, Investigation, Formal analysis.

## Ethical Form

Please note that this journal requires full disclosure of all sources of funding and potential conflicts of interest. The journal also requires a declaration that the author(s) have obtained written and signed consent to publish the case report report/case series from the patient(s) or legal guardian(s).

The statements on funding, conflict of interest and consent need to be submitted via our Ethical Form that can be downloaded from the submission site www.ees.elsevier.com/mmcr. **Please note that your manuscript will not be considered for publication until the signed Ethical Form has been received.**

## Declaration of competing interest

Dr Krista Keller currently serves on the scientific advisory board for MiDog, LLC, however, was not serving in this capacity at the time that this case used this diagnostic technology.
